# Implementation Considerations, Not Topological Differences, Are the Main Determinants of Noise Suppression Properties in Feedback and Incoherent Feedforward Circuits

**DOI:** 10.1371/journal.pcbi.1004958

**Published:** 2016-06-03

**Authors:** Gentian Buzi, Mustafa Khammash

**Affiliations:** Control Theory and Systems Biology, Department of Biosystems Science and Engineering, ETH Zurich, Zurich, Switzerland; National Center for Biotechnology Information (NCBI), UNITED STATES

## Abstract

Biological systems use a variety of mechanisms to deal with the uncertain nature of their external and internal environments. Two of the most common motifs employed for this purpose are the incoherent feedforward (IFF) and feedback (FB) topologies. Many theoretical and experimental studies suggest that these circuits play very different roles in providing robustness to uncertainty in the cellular environment. Here, we use a control theoretic approach to analyze two common FB and IFF architectures that make use of an intermediary species to achieve regulation. We show the equivalence of both circuits topologies in suppressing static cell-to-cell variations. While both circuits can suppress variations due to input noise, they are ineffective in suppressing inherent chemical reaction stochasticity. Indeed, these circuits realize comparable improvements limited to a modest 25% variance reduction in best case scenarios. Such limitations are attributed to the use of intermediary species in regulation, and as such, they persist even for circuit architectures that combine both IFF and FB features. Intriguingly, while the FB circuits are better suited in dealing with dynamic input variability, the most significant difference between the two topologies lies not in the structural features of the circuits, but in their practical implementation considerations.

## Author Summary

Essential to the survival of biological organisms is their ability to decipher and respond accordingly to stress scenarios presented by a changing and often unpredictable environment. Cellular noise, present due to the inherently random nature of both the external and internal environments, can obfuscate and corrupt the information found in the environmental cues, thus necessitating the development of mechanisms capable of repressing the noise and recovering the true information. Understanding these noise suppressing mechanisms is an important step toward the general understanding of adaptation and survival in biology. Here we present ideas that have broad implications for the understanding of the role and prevalence of two such mechanisms: the feedback and incoherent feedforward motifs. Using computational and analytical tools commonly employed in engineering applications, we characterize the performance and limitations of these two motifs, as well as establish their equivalence in dealing this several types of noise. We show that the effectiveness and preference of one motif over the other lies mostly in the practical implementation details and not in their structural properties.

## Introduction

Biological organisms grow in environments that, apart from tightly controlled laboratory conditions, tend to be highly diverse, unpredictable and changing. Essential to their survival is the ability to adapt and perform tasks necessary for life in such environments. This often involves robustly maintain desirable concentrations of a variety of molecular species [1]. This task is made even more difficult by the fact that biological processes are inherently stochastic and individual components are often imprecise. To provide robustness to such uncertainty, two mechanisms seem to be often employed by biological systems as part of the solution: feedback (FB) motif and incoherent feedforward (IFF) motif [2], [3].

Many studies show that IFF architectures provide robustness to noise and environmental uncertainty. In particular, native and synthetic microRNA (miRNA) mediated IFF circuits have been shown to provide gene expression robustness to gene dosage and other perturbations in mammalian cells [4], [5], [6]. Numerical studies have shown the potential for these circuits to reduce some of the inherent stochasticity of gene expression as well [7]. In a FB architecture, feedback regulation can be performed directly by the species of interest or alternatively through an intermediary species which serves as a proxy. Theoretical and experimental studies of direct feedback implementations show that such circuits provide robustness to noise [8], [9]. This holds true for proxy-regulated FB implementations as well, but such implementations are shown to be inferior at reducing overall gene expression noise compared to those of direct regulation topologies [10]. Other synthetic implementations of proxy-regulated FB architecture in mammalian cells highlight the limitations of such topologies in gene dosage invariance [6]. Other studies suggest that such FB circuits can suppress heterogeneity in gene expression by linearizing dose-response curves [11].

There are several different possible topological implementations of both IFF and FB motifs. In this paper, we consider two of the most commonly implemented of these topologies (one for each motif) and investigate their ability to suppress both extrinsic sources of variability and inherent stochasticity of chemical reactions. We make use of the control theoretic concept of *gain*, numerical simulations and analytical results to arrive at a deeper understanding of these two topologies and to show that different biological roles are not dictated by the structural properties of these circuits but are rather a result of the particular implementations. We highlight the limitations of each architecture and the mechanisms that give rise to such limitations.

## Results

Given a species of interest *Y* and an intermediary regulator species *X*, feedback (FB) and incoherent feedforward (IFF) circuits can be used to maintain the population of the *Y* close to a desired concentration in the presence of noise due to variations in the inputs to the circuits or due to the inherent stochasticity of chemical reactions. Here we examine how each circuit accomplishes this goal and what are the limitations of each of the architectures. Is any of the circuits better suited for this task?

**Fig 1:**
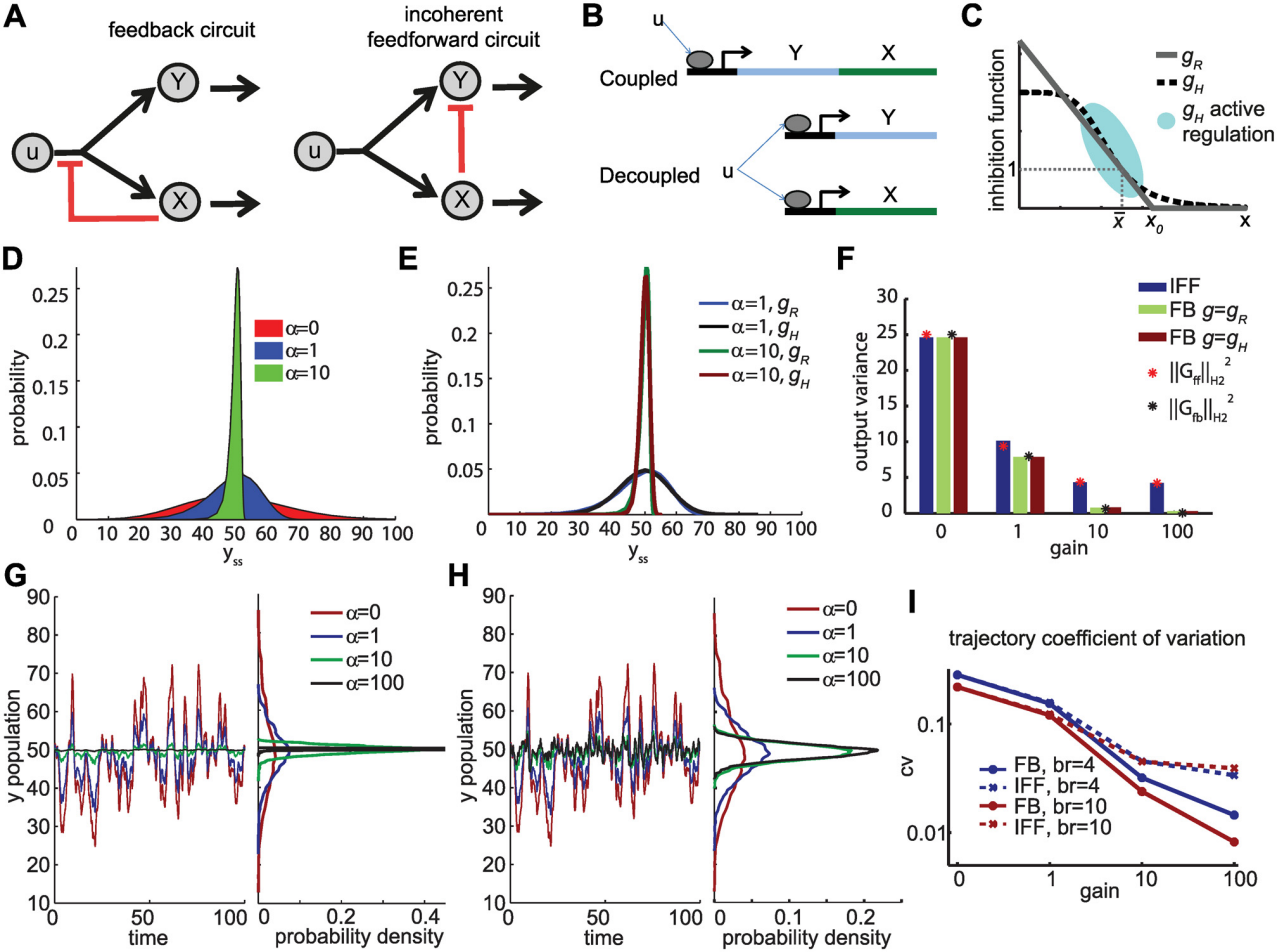
Adaptation in feedback (FB) and incoherent feedforward (IFF) architectures. **A.** Cartoon representation of FB and IFF architectures. Input *u* positively regulates species *X* and *Y* (black arrows). *X* inhibits the production of both *X* and *Y* in the FB architecture (red line, left panel) and mediates the degradation of *Y* (negative regulation) in the IFF architecture (red line, right panel). Both *X*, *Y* degrade at rates proportional to their respective populations (black arrows). **B.** Illustration of coupled and decoupled production. *X* and *Y* represent genes whose expression is under the control of the same promoter, but their production is considered coupled only if they are on the same transcript. **C**. Product inhibition functions considered in the FB implementations. **D**. The distribution of *y* at steady state for different values of regulation strength *α*. As the strength of regulation *α* increases, the distribution of y gets tighter around the nominal value $\bar{y}=50$. *u* is a Poisson distribution with mean $\bar{u}=10$. The distribution is the same distribution for both FB and IFF, with *g* = *g*_*R*_ used for FB.**E**. The distribution of *y* at steady state for different classes of inhibition functions, *g*_*R*_ and *g*_*H*_. **F**. Circuit response to white noise. ${\|G\|}_{H_{2}}^{2}$ is shown by black stars (for FB) and red stars (for IFF). The bars show the corresponding variance of *y*(*t*) of 10000 sample runs at *t* = 20. The noise in *u* modeled by adding $\sqrt{50}{\left[1,1\right]}^{T}\dot{w}$ to the right-hand side of the ODE models, where $\dot{w}$ is the standard Brownian motion.**G, H** Circuit response to input *u* given by a birth-death process (with birth rate *b*_*r*_ = 10 and death rate 1). Left panel shows sample trajectory in response to *u* for different gain implementations. Right panel shows the empirical probability distribution derived from each sample trajectory until final time *t* = 1000. Panel G shows the response of FB implementations using *g* = *g*_*H*_ and panel H that of IFF implementations. **I**. Log plot of the coefficient of variation (cv) of sample*y*-trajectories in response to *u* given by a birth-death process with birth rate *b*_*r*_ and death rate 0.1*b*_*r*_ (*t* ∈ [0, 1000]). Parameters used in D-I: $\bar{u}=10$, *k*_1_ = *l*_1_ = 5, *l*_2_ = 1, *f*(*u*) = 5*u*. For $g_{H}≔\frac{11}{1+10{\left(x/10\right)}^{n}}$, *n* = 1.1 (for *α* = 1), *n* = 11(for *α* = 10), *n* = 110 (for *α* = 100).

The specific FB and IFF architectures we consider consist of two species *X* and *Y* whose production is regulated by the same input signal *u*. Examples include genes that share the same promoter, mRNA and microRNA (miRNA) encoded in the same transcript, or proteins that are activated by the same environmental cues/signals. Because of the co-regulated nature of the production, the concentration of species *X* contains information about the concentration of species *Y* and can be used as a proxy for *Y*, analogous to how a reporter gene contains information about a gene of interest it has been transcriptionally fused to. The knowledge about *Y* thus contained in *X* can be used to control the concentration of *Y* when direct access to *Y* is not possible or impractical. When such knowledge is used to control *Y* by inhibiting its production, we get a FB circuit (Fig 1A, left). When it is used to control *Y* by either inhibiting *Y* directly or enhancing its degradation, we get an IFF circuit (Fig 1A, right). What makes this topology an IFF motif is that *u* both up-regulates and down-regulates *Y* (directly or through *X* respectively). The reaction schemes for both are shown in Table 1. For each circuit, there are two different implementations to consider, depending on whether the production of *X* and *Y* is coupled (*X* is produced anytime *Y* is produced) or decoupled (*X* and *Y* are produced by different reactions but with the same reaction rates). Fig 1B gives an illustrative example of differences between the two types of production: in both cases *X* and *Y* represent genes whose expression is under the control of the same promoter, but their production is considered coupled only if they are on the same transcript. We denote by *x* and *y* the population abundance (or concentration) of species *X* and *Y* respectively. For the FB circuit, *X* and *Y* have natural degradation rates *l*_1_*x* and *l*_2_*y* respectively (*l*_1_, *l*_2_ > 0) and production rate *f*(*u*)*g*(*x*) where *g*(*x*) (the inhibition function) is nonnegative monotone decreasing in *x* and *f*(*u*) is positive monotone increasing in *u*. Similarly for the IFF circuit, *X* and *Y* have natural degradation rates *k*_1_*x* and *k*_2_*y* respectively (*k*_1_ > 0, *k*_2_ ≥ 0) and production rate *f*(*u*). Furthermore *X* mediates the degradation of *Y* with rate *k*_12_ *xy* (*k*_12_ ≥ 0).

**Table 1:** Feedback and incoherent feedforward models.

	feedback		incoherent feedforward	
	coupled	decoupled	coupled	decoupled
degradation reactions	$X\;\overset{\;l_{1}x\;}{\to }\;\emptyset ,\;Y\;\overset{\;l_{2}y\;}{\to }\;\emptyset$		$\begin{array}{c} X\;\overset{\;k_{1}x\;}{\to }\;\emptyset ,\;Y\;\overset{\;k_{2}y\;}{\to }\;\emptyset \\ X+Y\;\overset{\;k_{12}xy\;}{\to }\;X \end{array}$	
production reactions	$\emptyset \;\overset{\;f(u)g(x)\;}{\to }\;X+Y$	$\begin{array}{c} \emptyset \;\overset{\;f(u)g(x)\;}{\to }\;X,\\ \emptyset \;\overset{\;f(u)g(x)\;}{\to }\;Y \end{array}$	$\emptyset \;\overset{\;f(u)\;}{\to }\;X+Y$	$\begin{array}{c} \emptyset \;\overset{\;f(u)\;}{\to }\;X,\\ \emptyset \;\overset{\;f(u)\;}{\to }\;Y \end{array}$
ODE model	$\begin{array}{c} \dot{x}=f\left(u\right)g\left(x\right)-l_{1}x\\ \dot{y}=f\left(u\right)g\left(x\right)-l_{2}y \end{array}$		$\begin{array}{c} \dot{x}=f\left(u\right)-k_{1}x\\ \dot{y}=f\left(u\right)-k_{12}xy-k_{2}y \end{array}$	
Normalization Condition	$l_{1}=k_{1},\;l_{2}=k_{2}+k_{12}\bar{x},\;g\left(\bar{x}\right)=1$			

We consider two main sources of noise that result in variations in *y*: variations/changes in the input *u*, and inherent stochasticity of the chemical reactions that compose the circuits themselves. We are going to distinguish between two different types of variations on *u*. The first kind is when *u* varies from cell to cell, but it changes very little over the lifetime of the cell. Such scenario might arise if *u* depends on plasmid abundance in the cell. Different cells have different plasmid concentrations and these concentration tend to change slowly over time, as is the case when plasmid are introduced into mammalian cells by transient transfection. A second kind of variation arises when *u* is dynamic and its fluctuations over the lifetime of the cell are not negligible. For example *u* can represent a dynamic signal that cells are exposed to (such as environmental stress signals) or depend on a dynamic molecular species concentration.

Next, we investigate the ability of each circuit to reduce variations in the output due to each of the sources. We examine each circuit under conditions for which each source of noise can be studied in isolation (i.e., one of the sources is dominant). This allows us to characterize analytically the capacity and limitation of each of these circuit to suppress each source of noise separately. We then look at the more general case when both contributions to output variance are comparable.

### Suppression of Variations Due to Input Noise

Consider the case when the number of molecules of *X* and *Y* is high and the inherent stochasticity of the chemical reactions is negligible (i.e., the dynamics of the loops are close to their macroscopic deterministic counterparts). We treat *x* and *y* as deterministic variables and examine how well the circuits respond to variations in *u*. Under these conditions, the coupled and decoupled implementations are indistinguishable and yield the same ODE model, shown in Table 1. In order to compare the performance of the two models in a meaningful way, we normalize them using the following criterion: for a given nominal value $\bar{u}$ of *u*, the production rates and the expected values of *x* and *y* at steady state (denoted by $\bar{x}$ and $\bar{y}$ respectively) must be the same in both models. This normalization, which results in the normalization conditions listed in Table 1, allows for a fair comparison since enforces both architectures to make use of the same resource amounts. Additionally, it provides a level of robustness of the results to the choice of noise measure [12]. We define a measure of the strength of regulations (i.e. how much control *X* has on *Y*) at steady state for each circuit by

$$\begin{array}{l} \alpha_{fb}≔-\bar{x}\frac{\partial g}{\partial x}(\bar{x})\\ \alpha_{ff}≔\bar{x}\frac{k_{12}}{k_{2}}. \end{array}$$ (1)

*α*_*fb*_ is the “effective feedback gain” of feedback and is given by the slope of the inhibition function—see S1 Text for more details. *α*_*ff*_ is the “effective feedforward gain” of feedforward regulation (see S1 Text) and is given by the ratio of the mediated degradation of *Y* to the unmediated degradation of *Y*. Let *y*_*ss*_(*u*) be the steady state concentration of *y* at any input value *u*. We are interested in quantifying how variations in *u* affect *y*_*ss*_.

#### Cell to cell input variability

The first case we consider is when the variability in *u* comes from cell to cell variability, i.e., each cell has a different *u* but such *u* is constant over time. How do differences in *u* affect the steady state expression levels of *y* for different cells? For example, how does cell *A* having *δu* more plasmids then cell *B* manifest itself in the steady state expression levels of *Y* (denoted by *y*_*ss*_) in cell *A* versus cell *B*? An answer to this question can be provided by the *sensitivity* of *y*_*ss*_ with respect to *u*, denoted by $\frac{\partial y_{ss}}{\partial u}$, which describes how small changes in *u* are transmitted into changes in *y*_*ss*_. For both circuits, this sensitivity near $\bar{u}$ is given by

$$\begin{array}{c} \frac{\partial y_{ss}}{\partial u}\left(\bar{u}\right)=\frac{1}{1+\alpha }\frac{f^{\prime }\left(\bar{u}\right)}{f(\bar{u})}\bar{y} \end{array}$$ (2)

where *α* = *α*_*fb*_ for the FB and *α* = *α*_*ff*_ for the IFF. It is clear from Eq (2) that higher effective gains *α*_*fb*_ and *α*_*ff*_ (i.e., more aggressive control) make *y*_*ss*_ less sensitive to cell to cell variations in *u* and for high enough effective gains the effects of such variation can be suppressed almost completely. It should be noted that $\frac{\partial y_{ss}}{\partial u}(\bar{u})$, though a local measure of sensitivity, it can still provide an overall picture of the sensitivity of the system to changes in *u* by varying $\bar{u}$ and recalculating. A more direct way is to explicitly calculate the dependence of *y*_*ss*_ to *u*. For the FB an explicit formula is not possible for the most general classes of inhibition functions *g*. Consider a specific class of inhibition functions *g* given by *g*_*R*_

$$\begin{array}{c} g_{R}\left(x;\alpha_{fb}\right)≔\max\left\{1+\alpha_{fb}-\alpha_{fb}\frac{l_{1}}{f(\bar{u})}x,0\right\}, \end{array}$$ (3)

i.e., *g*_*R*_ is a nonlinear function composed of a linearly decreasing part for $x<x_{0}≔\frac{1+\alpha_{fb}}{\alpha_{fb}}\frac{f(\bar{u})}{l_{1}}$ and an identically 0 part for *x* > *x*_0_ (i.e., a ramp—see Fig 1C, solid gray line). Notice that this parametrization of the inhibition function *g* is consistent with the definition of *α*_*fb*_ in Eq 1. For this choice of *g* and with the normalization applied, both circuit topologies produce the same functional form for the dependence relation:

$$\begin{array}{c} y_{ss}=\frac{\left(1+\alpha \right)f\left(u\right)}{\left(1+\alpha \frac{f(u)}{f(\bar{u})}\right)}\frac{\bar{y}}{f(\bar{u})}, \end{array}$$ (4)

where *α* = *α*_*FB*_ for the FB and *α* = *α*_*FF*_ for the IFF. This implies that for the same level of regulation, both circuits have the same ability to adapt to variations in *u*, and that stronger regulation ensure better adaptation. As an illustration, consider the following numerical example. Let the distribution of *u* from cell to cell be a Poisson distribution with mean 10, and let *f*(*u*) = 5*u*, *l*_1_ = 5, *l*_2_ = 1. Fig 1D shows the distribution of *y*_*ss*_ for different values of *α* (same distribution for both FB and IFF). As the strength of regulation *α* increases, the distribution of *y*_*ss*_ gets tighter around the value $\bar{y}=50$ (nominal *y*_*ss*_), which means that even large cell to cell variations of *u* will result in small variations of *y*_*ss*_.

It is important to note that the goal of using *g*_*R*_, here and in the rest of this paper, is to analytically derive properties of the feedback architecture, not necessarily as a computational substitute of other classes of inhibitions functions. At the same time, use of *g*_*R*_ is not merely an analytical/computational tool, but there is strong evidence of systems, such as cAMP regulation of carbon uptake in *E. coli*, that are best described by this type of inhibition function [13]. In this systems, the data in the data range produced by the experiments is best described by a linear inhibition function (Fig 2A in [13]), which matches the linear part of a ramp, and with no available data outside of the linear range of the ramp. For more general classes of inhibition functions *g*, the results can numerically be shown to be similar to that of *g* = *g*_*R*_, as long as variations in *u* do not push the operating point of the feedback away from the active region of regulation (the values for which the graph of *g* is not flat—see Fig 1C). For example, when *g* is parameterized by a Hill type functions $g_{H}≔\frac{1+k_{x}}{1+k_{x}{\left(x/\bar{x}\right)}^{n}}$, the differences in the resulting distributions of *y*_*ss*_ for *g* = *g*_*H*_ and *g* = *g*_*R*_ are minor (Fig 1E) and the same general conclusions about system behavior hold. Unless otherwise stated, in this paper we use the parametrization *g* = *g*_*R*_. Notice that this is not an linearization of a general nonlinear system, but a specially parameterized nonlinear system. In most numerical simulations we also test the validity of our results by using Hill-type functions (*g* = *g*_*H*_) to provide evidence that the nature of the results is not dependent on the particular choice of the class of inhibition functions.

Eqs (2) and (4) imply that both FB and IFF circuits have the same ability to suppress this type of extrinsic variability. Furthermore, for the same strength of regulation (*α*_*fb*_ = *α*_*ff*_) their performance based on this criteria is indistinguishable. They also suggest that performance for both systems can be improved by faster reactions, i.e., by having higher inflow (production rates) and by implication higher outflow (total degradation rates). The special case of *k*_2_ = 0 (*α*_*ff*_ = ∞) the IFF model, studied in detail in [14], displays perfect adaptation. This is indicative of integral-like feedback [15], and implies that for large but finite *α*_*ff*_ (i.e., *k*_2_ > 0 small) the IFF can be viewed as leaky integral control. It should be mentioned that for the IFF implementation considered in the main text, the role of the proxy *X* in the mediated degradation of the species of interest *Y* is purely catalytic (i.e. the species *X* is not consumed). However, even if the effect is not purely catalytic, the nature of this section’s results does not change (see S1 Text).

#### Dynamic input fluctuations

Consider the case when *u* is dependent on a dynamic signal, i.e., $u\left(t\right)=\bar{u}+\delta \left(t\right)$ where *δ*(*t*) is a mean 0 process, which can be deterministic or stochastic. For small magnitude *δ* and when *y*(*t*) is close to $\bar{y}$, how fluctuations in *u* are reflected in fluctuations in *y* is captured in the frequency domain by a function *G* (called a *transfer function*, see S1 Text for details). This type of approach of analyzing the effects of noise/disturbances by examining the signal frequency components has been successfully used in several biological systems such as cell lineage pathways [16], the glycolysis pathway [17] and the hyperosmolar signaling pathway in yeast [18]. A quantity of interest is the so-called $H_{2}$-norm of *G*, denoted by $\|G{\|}_{{ℋ}_{2}}$, whose square is equal to the variance of *y*(*t*) as *t* → ∞ when *δ* is given by the standard Brownian motion [19]. Analysis of *G* and $\|G{\|}_{{ℋ}_{2}}$ reveals that regulation (especially aggressive regulation) can reduce the variance of y due to the variance of *u*, but also that the FB topology is better suited for the task. Indeed, high gain FB implementations can drive the variance of *y*(*t*) to zero while for any IFF implementation there exist a lower bound on the variance of *y* given by $\frac{1}{2(l_{1}+l_{2})}$ (see S1 Text for details). This is illustrated in Fig 1F, where the noise in *u* is modeled by adding a scaled Brownian motion term to the right-hand side of the equations in ODE models in Table 1. The theoretical predictions based on $\|G{\|}_{{ℋ}_{2}}$ are shown by red and black stars, while the bars show the corresponding variance of *y*(*t*) for sample runs of implementations with different regulation strengths *α*. The figure shows that $\|G{\|}_{{ℋ}_{2}}$ can be a good predictor of the variance of *y* even in the full system implementation and such predictions are not sensitive to the choice of the class of the inhibition function *g*. We should note that the information content provided by *G* is not dependent on the nature of *δ*(*t*), —i.e., whether *δ*(*t*) is given by the Brownian motion or even if it is not stochastic at all -, and the results do not depend on the process assumptions on *δ*. In fact, *G* captures how each individual frequency component of *δ*(*t*) is attenuated or amplified by the circuit. Fig 1G and 1H shows how different implementations of the circuits respond when *u* is given by a birth-death process. The corresponding means and standard deviations of the sample trajectories are also shown. The presence of even low levels of regulation does reduce the variations on *y*-trajectories for both circuits. In general, for different *u*(t) the high gain FB implementations outperform their IFF counterparts as illustrated in Fig 1I. The figure shows the coefficient of variation of typical sample trajectories in response to *u*, where *u* is given by a birth death process with birth rate *b*_*r*_ and death rate 0.1*b*_*r*_. An intuitive explanation of why FB outperforms IFF, is that both steady state robustness and the transient dynamics matter under these conditions. In both topologies the transient dynamics of *y* depend on *x*, but it is only the FB implementations that regulate the dynamics of *x* in addition to those of *y*. It is this additional robustness of the intermediary species *X* that accounts for the better performance.

### Suppression of Chemical Reaction Stochasticity

In this section *x* and *y* are random variables that refer to the population of *X* and *Y* respectively. We study how different effective gain *α*_*fb*_ and *α*_*ff*_ change the noise properties of FB and IFF respectively, by looking at the variance of the stationary distribution of *y*. Throughout the rest of this section we will assume that the value of the input *u* is fixed at $u=\bar{u}$. The stochastic models are constructed based on the reaction schemes shown in Table 1. Similar to the previous section, in order for the comparisons between the different topologies to be meaningful, we require that the different implementations have the same expected steady state values (steady state means) for both *x* and *y* and same propensities for the production reactions at the steady state mean values. We denote by < ⋅ > and *var*(⋅) the expected value and the variance with respect to the stationary distribution.

#### Feedback architecture

Consider the case when for a given *α*_*fb*_, the inhibition function *g* is parametrized by Eq (3) (i.e., *g* = *g*_*R*_). We consider the set of feedback gains *α*_*fb*_ for which $x_{0}=\frac{1+\alpha_{fb}}{\alpha_{fb}}\frac{f(\bar{u})}{l_{1}}$ is an integer. For this particular choice of *g*, we can analytically compute the exact steady state mean and variance of *x* and *y* for both coupled and uncoupled stochastic model of the FB architecture using the moment equations.

**Fig 2:**
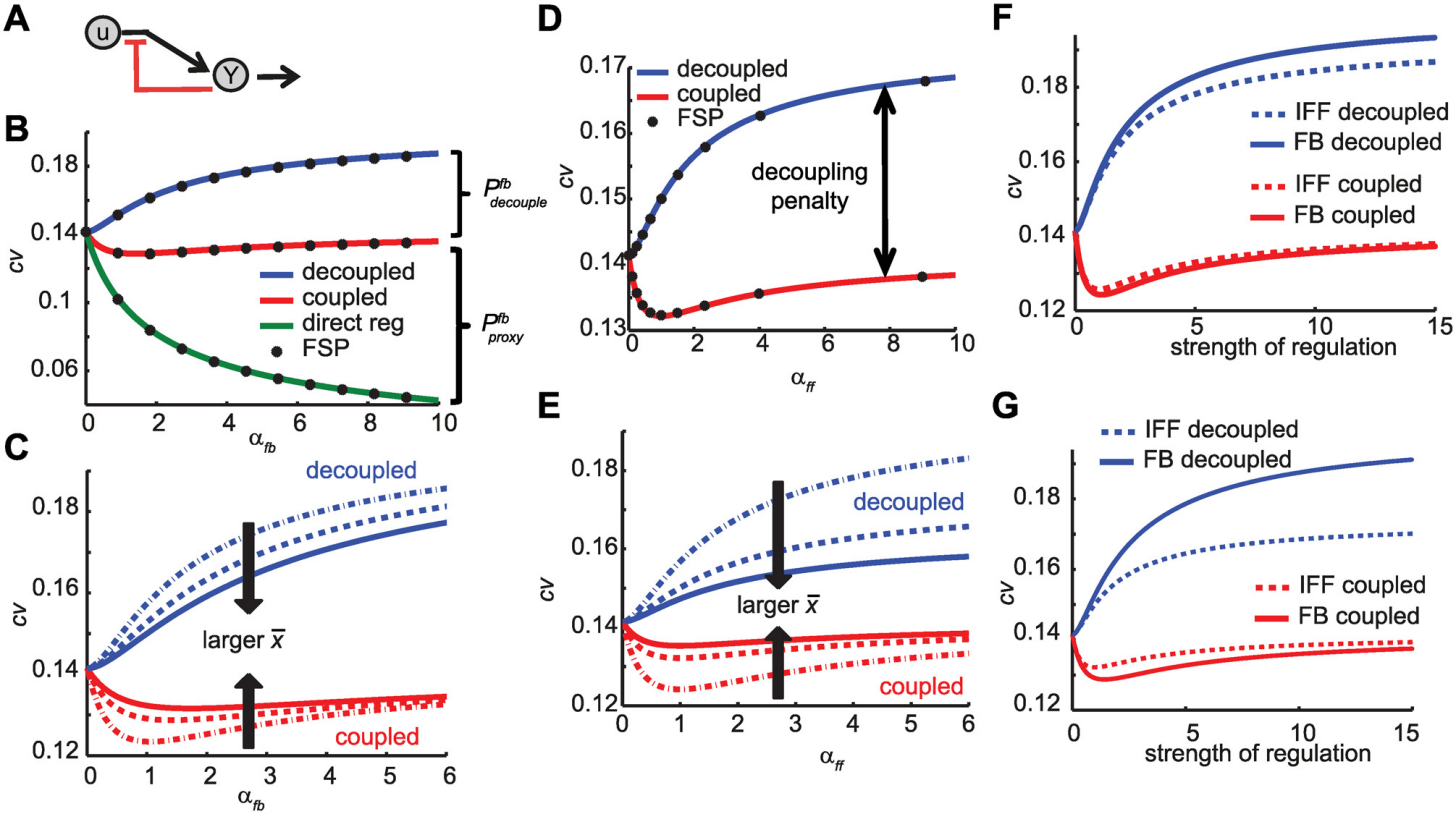
Suppression of intrinsic fluctuations in FB and IFF architectures. **A**. Cartoon representation of direct feedback regulation architecture. **B, C**. Suppression of inherent stochastic fluctuation in FB architecture. Shown is the coefficient of variation (cv) as a function of the effective gain *α*_*fb*_. (B) Direct regulation (green line) can completely suppress the fluctuations for high enough gains. The coupled implementations (red line) can reduce the cv, but such reductions are moderate and best for small gains. The decoupled implementation always increases the cv. Shown are also the penalty terms $P_{couple}^{fp}$, $P_{proxy}^{fp}$. Parameters used: $\bar{y}=50$, $\bar{x}=50$. Shown are FSP solutions (black dots) for implementations with *f*(*u*) = 50, *l*_1_ = 1, *l*_2_ = 1$g\left(x\right)=\frac{11}{1+10{\left(x/50\right)}^{h}}$, *h* = 0, 1, …, 10). (C) The benefits of coupled implementation (red lines) and the penalties of the decoupled implementation (blue lines) are reduced as $\bar{x}$ increases (compared to $\bar{y}$). Shown is cv for $\bar{y}=50$, $\bar{x}=5,50,100$. **D, E**. Suppression of inherent stochastic fluctuation in IFF architecture. Shown is the cv as a function of the effective gain *α*_*ff*_. (D) The coupled implementations (red line) can reduce the cv, but such reductions are moderate and best for small gains. The decoupled implementation always increases the cv. Parameters used $\bar{y}=50$, $\bar{x}=50$. Shown are FSP solutions (black dots) for implementations with *f*(*u*) = 50, *k*_1_ = 1, and *k*_2_ and *k*_12_ as shown in Table A in S1 Text. (E) The benefits of coupled implementation (red lines) and the penalties of the decoupled implementation (blue lines) are reduced as $\bar{x}$ increases. Shown is cv for $\bar{y}=50$, $\bar{x}=5,50,100$. **F, G**. Comparison of FB and IFF architectures. For the same gains, FB coupled implementation (solid red line) performs slightly better than the IFF counterpart (dotted red line), and the FB decoupled implementation (solid blue line) performs slightly worse than the IFF couterpart (dotted blue line). Such differences are emphasized for smaller $\bar{x}$. Shown are implementations with $\bar{y}=50,\bar{x}=50$ (panel E) and $\bar{y}=50,\bar{x}=10$ (panel F).

In order to better understand the various terms in the expressions for the variances, we first examine an even simpler case of feedback inhibition architecture. Consider the case when *Y* directly inhibits its own production (Fig 2A) with inhibition function *g*(*y*) = *g*_*R*_(*y*;*α*_*fb*_) (i.e., the reaction scheme is given by $\emptyset \;\overset{\;f\left(u\right)g_{R}\left(y\right)\;}{\to }\;Y,\;Y\;\overset{\;l_{2}\;}{\to }\;\emptyset$). This architecture is similar to that of [9] with the burst size exactly 1. At steady state *y* is distributed according to a binomial distribution (see S1 Text, Lemma 1) with mean $\bar{y}≔<y>=\frac{f(\bar{u})}{l_{2}}$ and variance $var\left(y\right)=\frac{1}{1+\alpha_{fb}}\bar{y}$. This implies that high feedback gain *α*_*fb*_ always improves the variance and very large *α*_*fb*_ can almost completely suppress stochastic fluctuations. This is to be expected since *Y* directly inhibits its own production and for large *α*_*fb*_ and *y* near $\bar{y}$, it is unlikely for either one of the production or degradation reactions to fire twice in a row (since $y>\bar{y}$ implies that degradation reaction is much more likely to fire and $y<\bar{y}$ implies the production reaction is much more likely to fire).

Consider now the original FB architecture (Fig 1A left). In the case when the production of *X* and *Y* is coupled, *y* has the same steady state mean $\bar{y}=\frac{f(\bar{u})}{l_{2}}$, but an additional penalty term appears on the expression for the steady state variance of *y*

$$\begin{array}{c} \begin{array}{c} var\left(y\right)=\frac{1}{1+\alpha_{fb}}\bar{y}+P_{proxy}^{fb} \end{array} \end{array}$$

where

$$\begin{array}{c} P_{proxy}^{fb}=\frac{\alpha_{fb}\left(\alpha_{fb}+\bar{x}/\bar{y}\right)}{\left(1+\alpha_{fb}\right)\left(\left(1+\alpha_{fb}\right)+\bar{x}/\bar{y}\right)}\bar{y}, \end{array}$$ (5)

and $\bar{x}≔<x>=\frac{f(\bar{u})}{l_{1}}$. $P_{proxy}^{fb}$ is the “price” paid for using the population abundance of *X* to estimate that of *Y*. As a consequence, larger values of *α*_*fb*_ do not guarantee improvement in the variance of *y* (Fig 2B). In fact, because of the “proxy penalty” relatively small gain implementations minimize the variance. Such improvements are biggest when the ratio $\frac{\bar{x}}{\bar{y}}$ is smallest (Fig 2C). An intuitive explanation is that a proxy in low copy numbers senses better the fluctuations in the firing rate of the production reaction, and consequently makes the feedback controller better at responding to those fluctuations. In fact when $\bar{x}$ is very large ($\frac{\bar{x}}{\bar{y}}\to \infty$) any gain produces the same variance as the uncontrolled case (*α*_*fb*_ = 0). When $\bar{x}$ is comparable to $\bar{y}$ ($\frac{\bar{x}}{\bar{y}}=1$), the most feedback can reduce the steady state variance is by≈17% compared to the uncontrolled case. The biggest achievable reduction in variance is 25%, when $\bar{x}$ is very small compared to $\bar{y}$ ($\frac{\bar{x}}{\bar{y}}\to 0$).

In the case when the production of *X* and *Y* is decoupled, we get the same values for $\bar{y}$ as in the coupled case, but a decoupling penalty term appears on the expression for the steady state variance of *y*

$$\begin{array}{c} var\left(y\right)=\frac{1}{1+\alpha_{fb}}\bar{y}+P_{proxy}^{fb}+P_{decouple}^{fb} \end{array}$$

where

$$\begin{array}{c} P_{decouple}^{fb}=\frac{\alpha_{fb}}{1+\alpha_{fb}+\bar{x}/\bar{y}}\bar{y}. \end{array}$$ (6)

In this case, the best implementation is that of gain $\alpha_{fb}^{opt}=0$ (i.e., no feedback). Therefore, when the productions of *X* and *Y* are separate reactions, any feedback would result in higher steady state variance of *y* (Fig 2B). This indicates that when decoupled in production, fluctuations in *x* do not provide useful information on the fluctuations on *y* and any action on that information content would amplify the noise. Unlike the proxy penalty term, $P_{decouple}^{fb}$ gets larger for smaller ratios $\bar{x}/\bar{y}$ causing the variance of the decoupled system to increase as the $\bar{x}$ gets small (Fig 2C).

The above results extend well numerically even for different classes of inhibition functions *g*. Consider the case when *g* is given by a Hill type function *g*_*H*_. We use finite state projection (FSP) solutions [20] with *g* = *g*_*H*_ to compare the steady state values of the moments of *x* and *y* to those of analytical results derived using *g* = *g*_*R*_, with the same slope at the steady state value ($\bar{x}=\frac{f(\bar{u})}{l_{1}}$). The numerical solutions show that the moment values derived using *g* = *g*_*R*_ capture the overall trend and are a good approximation for the values with *g* = *g*_*H*_ as shown in Fig 2B. FSP solutions provide strong evidence that for these models, *g*_*R*_ captures the features of the network and the results derived using *g*_*R*_ extend to other types of monotone decreasing inhibition functions *g*.

#### Incoherent feedforward architecture

For the IFF stochastic models based on the reaction scheme in Table 1, the lower order moments dynamics of *y* depend on the higher order moments dynamics. As a consequence it is not possible to get exact analytical expressions for the steady state mean and variance of *y*. However, one can get approximate expressions for the moments by approximating the higher order moment terms in the moment equation using approximations schemes known as moment closure methods [21]. Here we use one such method called derivative matching [22] to get analytical expressions for the approximated mean and variance of the stationary distribution of *y*.

First, we consider two special cases: (1) no regulation (*k*_12_ = 0, *α*_*ff*_ = 0) and (2) infinite gain regulation (*k*_2_ = 0, *α*_*ff*_ = ∞, equivalent to integral control and perfect adaptation in the deterministic case). For no regulation case *α*_*ff*_ = 0, both the coupled and decoupled production topology result in $var\left(y\right)=\bar{y}$ where $\bar{y}=\frac{f(\bar{u})}{k_{2}}$ (*y* is a simple birth death process). For the second case *α*_*ff*_ = ∞, for a given mean $\bar{y}$, $var\left(y\right)=\bar{y}$ for the coupled topology and $var\left(y\right)=\bar{y}+P_{decouple,\infty }^{ff}$, where $P_{decouple,\infty }^{ff}≔\frac{\bar{y}+1}{1+\bar{x}/\bar{y}}$ is a decoupling penalty term. The penalty term gets worse as $\bar{x}\to 0$, causing the variance to more than double. As $\bar{x}\to \infty$ (*x* behaves more deterministically) the penalty term vanishes.

For the general case (*k*_2_ > 0, *k*_12_ > 0), the explicit approximate analytic solutions for the mean and variance of *y* at stationary are too messy to be useful in providing any direct insight on how different gains and other parameter combinations affect performance. The behavior is similar to that of the FB mean and variance. For the coupled IFF, effective feedforward gains *α*_*ff*_ can reduce the variance (compared to *α*_*ff*_ = 0, no control), but such reduction is limited (maximum 30% improvement—indeed maximum 25% improvement when average populations are no smaller than 2) and best for small values of *α*_*ff*_ $(\alpha_{ff}^{opt}=\frac{\bar{x}+\bar{y}}{1+\bar{x}+\bar{y}}$). In the case of the decoupled IFF, any regulation (*α*_*ff*_ > 0) increases the variance (Fig 2D). [7] have made similar observation by exploring the parameter space of a different model implementation of miRNA mediated feedforward loops using numerical simulations and approximate analytical formulas (derived through linearization of the propensity functions).

For a fixed $\bar{y}$, smaller $\bar{x}$ improves the performance of the coupled IFF (reduces variance), but hurts the performance of the decoupled IFF (Fig 2E). This type of behavior is not surprising. Similar to the FB case, for the coupled IFF low copy numbers of *X* means IFF senses better the fluctuations in the firing rate of the production reaction and consequently responds better to those fluctuations. However, when *X* and *Y* are decoupled, IFF is simply responding to unrelated variation in *x* due to the inherent stochasticity of chemical reactions (which is larger for low copy numbers of *X*). FSP solutions of the stochastic models show the steady state values of the moments derived using derivative matching are good approximation to the actual values of these moments (Fig 2D).

#### Comparing the different architectures

Both FB and IFF circuits respond in a similar manner to chemical reaction stochasticity and achieve comparable reductions in steady state variance (no more than 25% in all but a few extreme scenarios). Direct comparison of coupled FB and IFF reductions for optimal values of gains, shows that for average molecule counts of *X* and *Y* that are no smaller than 2, the FB realizations achieve larger reductions than the IFF counterparts. The larger the average population of *X*, the more FB outperforms the IFF. Notice also that the optimal gain for the FB is larger than the optimal gain for the IFF.

We already gave some intuitive reasons on why such hard limitations on the ability of these circuits to deal with inherent chemical stochasticity of the reactions. To gain some more insight, we approximate the stochasticity of each reaction by a Brownian motion term using Linear Noise Approximation (LNA) [23] and look at the $H_{2}$-norm of the corresponding transfer function (see S1 Text for details). This way we can decompose the total variance based on the contributions of each individual reaction, and evaluate the limitation imposed on the controller. A first observation is that 50% of the (uncontrolled) variance is contributed from reactions that are independent of *α*, so the controller can only improve on the remaining 50%. Another observation is that any action on information content from uncorrelated noise sources will amplify the variance, while action on correlated noise will reduce the variance. For the decoupled topology the controllers are acting on two sources of uncorrelated noise (both production and degradation of *X* are uncorrelated with that of *Y*), hence any action will result in an increase of the overall variance. For the coupled topology the controllers are acting on one source of correlated noise (production of *X* and *Y*) and one source of uncorrelated noise (degradation reactions), resulting on a tradeoff and values of *α* for which the controller is maximally beneficial will result in a maximum 50% reduction of the controllable portion of the variance (25% overall). (see S1 Text for more details).

### Concurrent Suppression of Both Sources of Variability

So far we have shown that if the stochasticity of the chemical reactions can be ignored (i.e., the dynamics of the circuit can be faithfully represented by their macroscopic representation), regulation always reduces variations due to input noise or even completely suppress such variations for strong enough levels of regulation. On the other hand, if the input noise can be ignored and the primary source of noise is the randomness of the chemical reactions, regulation (especially strong regulation) is not always beneficial and at best can only reduce the variance by 25%. However such reductions of the extrinsic and intrinsic noise cannot be achieved simultaneously by the same circuit realization since such improvements require different effective gains which are hard-encoded by the reaction propensities. How aggressively the circuits should regulate the production (for the FB) or the degradation (for the IFF) of *Y* for noise suppression is determined by the tradeoff between suppressing the various sources of noise. We illustrate this idea with an example.

Consider three different FB circuit realizations: (1) no feedback regulation (*α*_*fb*_ = 0, *g*(*x*)≡1) (2) maximum level of regulation (very large effective gain, *α*_*fb*_ → ∞) with coupled production and (3) maximum level of regulation (*α*_*fb*_ → ∞) with decoupled production. For the last two realizations, the feedback inhibition function *g* is given by Eq (3). Let the input *u* be a random variable distributed according to a Poisson distribution with mean $\bar{u}>0$, and let *f*(*u*) = *au* + *b*.

The first realization (no feedback) results in

$$\begin{array}{ccc} var(y) & = & \underset{chemical\;reaction\;stochasticity}{\underset{︸}{\frac{C+b}{l_{2}}}}+\underset{input\;variability}{\underset{︸}{\frac{C^{2}}{l_{2}^{2}}\frac{1}{\bar{u}}}}, \end{array}$$

where $C≔a\bar{u}$. Each of the terms in the expression correspond to the contributions to the variance due to each noise source. For a fixed *C* and *b* (which guarantees fixed average inflow $f(\bar{u})$), smaller $\bar{u}$ implies larger total variance. This is to be expected since smaller $\bar{u}$ means that Poisson variable *u* becomes more “noisy”. Note that for this realization, both coupled and decoupled implementations yield the same *var*(*y*).

The second FB realization (coupled with very strong feedback) results in a smaller variance, $var\left(y\right)=\left(C+b\right)l_{2}^{-1}$ for any finite $\bar{u}$. This is a result of strong feedback completely suppressing the variance term due to stochasticity in *u* but not changing the variance term due to stochasticity of the chemical reactions. It is clear that this realization is preferable to no feedback for all parameter values.

The third FB realization (decoupled with very strong feedback) results in variance $var\left(y\right)=2\left(C+b\right)l_{2}^{-1}$. Strong feedback again completely suppresses the variance term due to randomness in *u* but it also doubles the variance term due to randomness of the chemical reactions. So this realization would be preferable to no feedback only if $\bar{u}<\frac{C^{2}}{l(b+C)}$, i.e. the input noise level is above a specific threshold.

**Fig 3:**
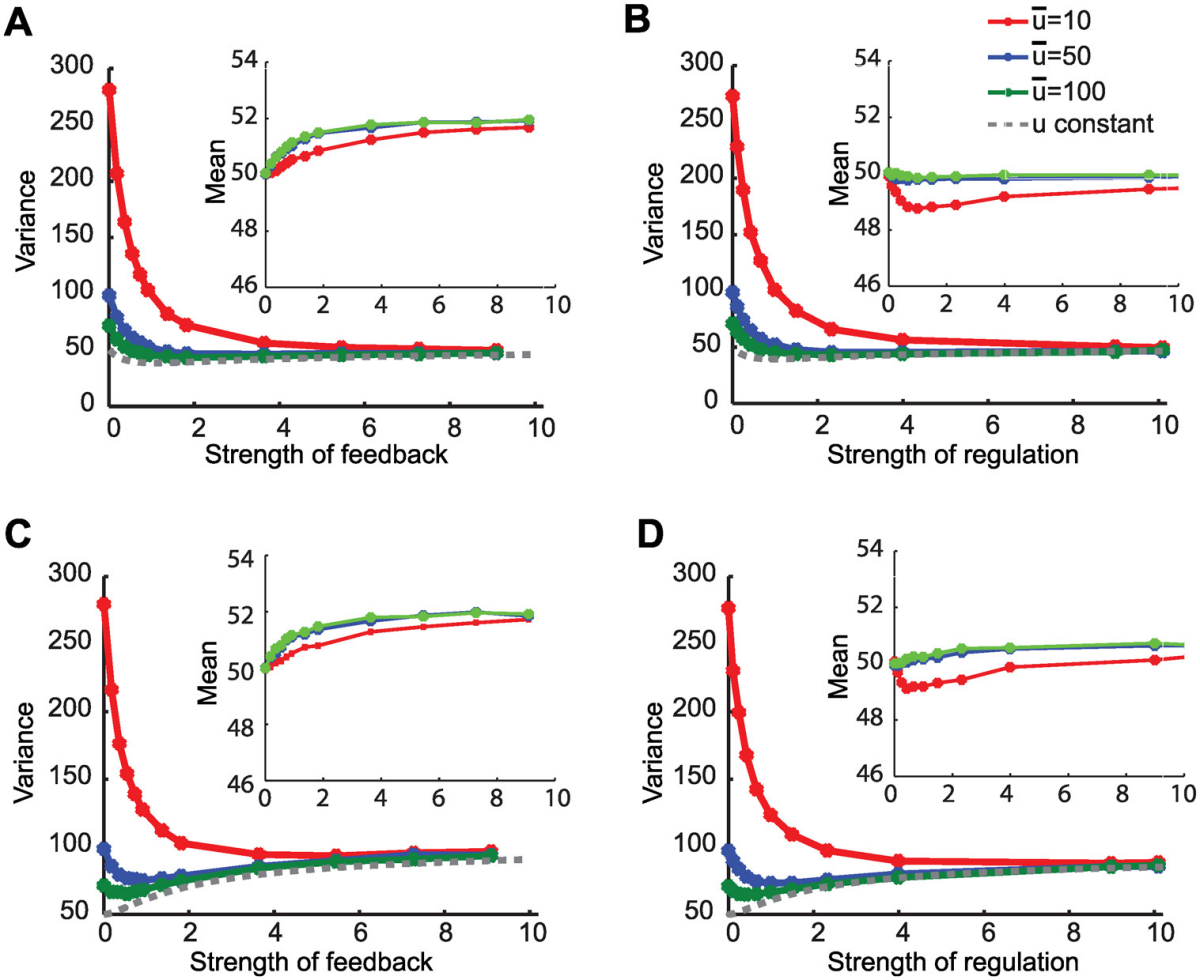
Simultaneous suppresion of both sources of fluctuations. For all panels, shown in the main window is the variance as a function of the strength of regulation and shown in the inset is the mean as a function of the strength of regulation. Low $\bar{u}$ implies noisier input *u* and higher extrinsic variability. When the dominant source of variability is input fluctuations, then a strong regulation strategy is preferable. However when the contributions of input fluctuations and chemical reaction stochasticity are comparable, then strategies with smaller gains are preferable. This is especially true for the decoupled implementations. **A**. Coupled FB implementation **B**. Coupled IFF implementation **C**. Decoupled FB implementation **D**. Decoupled IFF implementation. Values were obtained using 40,000 SSA simulations of models shown in Table B in S1 Text.

In general, for both FB and IFF circuits the best regulation strategy is dependent on the relative dominance of the noise sources. Fig 3 shows numerical stochastic simulation algorithm (SSA) results [24] for different circuit realizations and different input noise scenarios. As the input noise level is high, the noise term due to input noise is dominant and stronger regulation is preferred. For low levels of input noise, the noise term due to chemical reaction stochasticity becomes dominant and small levels of regulations are preferred especially for the decoupled realizations.

### Combining the FB and IFF into a Single Circuit

What happens if the intermediary species controls both the production and the degradation of *y* simultaneously? Would it result in better adaptation/noise suppression than the corresponding FB or IFF circuit? To answer this questions we consider here a combined architecture described in more detail in S1 Text. To fairly compare between all architectures, the same normalization criteria, captured by the conditions listed in Table 1, is applied and the performance of the combined circuits in response to the same sources of variability is examined.

If we define the “effective gain” *α*_*comb*_ of the the combined architecture by

$$\begin{array}{c} \alpha_{comb}≔\alpha_{fb}+\alpha_{ff}+\alpha_{fb}\alpha_{ff}, \end{array}$$ (7)

then we observe that in adapting to cell-to-cell variability, both eqs 2 and 4 hold, for *α* = *α*_*comb*_. Since adaptation to this type of variability is dependent on implementations with high effective gains circuits, the combined architecture offers the advantage that it can implement gains that might not be possible biologically in each of the FB or IFF circuits individually. Similarly, in suppression of dynamic input variability, the combined architecture also takes advantage of the best of both FB and IFF topologies in improving the performance (see S1 Text for details).

On the other hand the combined topology does not offer any extra benefits in suppressing inherent stochasticity of the chemical reactions. LNA approximation of the stochastic dynamics reveals that the maximum improvement achievable is still at 25% of the variance (as $\bar{x}/\bar{y}\to 0$, for optimal gains satisfying *α*_*comb*_ = 1). This theoretical result suggests that the limitations arise from the use of the intermediary (“a noisy sensor”) and not from the regulation strategy.

## Discussion

The feedback (FB) and incoherent feedforward (IFF) circuits studied here control the concentration of a species of interest (denoted *Y*) by making use of an intermediary species (denoted *X*) that serves as a proxy for *Y*. The two circuits differ in how the information contained in the proxy is used: FB uses it to control the production of *Y*, while IFF uses it to control its degradation. Employing a control theoretic approach, we quantified the strength of regulation of each circuit implementation by evaluating its “effective gain”, which measures how aggressively *X* regulates the production of *Y* (FB circuit), or its degradation (IFF circuit). When we compare implementations with the same effective gains we observe that both architectures have similar properties in adaptation and noise suppression. We considered three basic types of variability that the circuits need to deal with: static extrinsic cell-to-cell variability, dynamic input variability, and intrinsic chemical reaction stochasticity.

### FB and IFF circuits Are Equally Effective in Adapting to Cell-to-Cell Variability

This includes scenarios when static cell-to-cell variations, such as plasmid abundance variability in a cell, which result in variability to the input of the circuits. A common performance objective is the minimization of variability in steady-state expression levels of *Y* in the presence of such input variability. Under this objective, implementations of both architectures with the same “effective gains” perform identically. In particular both architectures can almost entirely suppress these types of variations in the input provided that high enough effective gain implementations are realizable.

Our study establishes that the main differences between the two motifs involves practical considerations, such as what effective gains are biologically feasible to implement in the system of interest and the cost of such implementations. Biological constructs, such as micro-RNA (miRNA) post-transcriptional regulation, make the implementation of high gain IFF implementations practical. Such implementations simply require high ratios of mediated degradation to the native degradation of the species of interest *Y*. Furthermore, these constructs allow for tunable synthetic implementation, which indeed have shown better adaptation for stronger mediated degradation (i.e., higher effective gain) [5]. On the other hand, high gain FB implementations require inhibition functions with high slopes in the operating regime which are generally harder to realize. The range of achievable gains in the operating regime of interest might account for why many synthetic implementations of IFF circuits provide adaptation while FB implementations do not [6].

### FB Circuits Are Better Suited to Deal with Dynamic Variations of the Input

This includes scenarios where the main source of variation is the dynamic nature of the input, such as when the input to the circuits is dependent of time-varying environmental stress signals. Under this type of variation, the variance over time of the expression level of the species of interest can be made as small as needed using high gain FB implementations. On the other hand, this is not true for IFF since there exists a lower bound for how small this variance can be made by any IFF implementation.

### FB and IFF Circuits Are Not Effective in Reducing Fluctuations Due to Inherent Chemical Reaction Stochasticity

The performance of same strength implementations of both circuits are comparable and both are severely limited on how much reduction in variance they can achieve. Such reduction is restricted to a maximum of 25% (when the expected abundance of both species is no smaller then 2), and is a result of the use of the intermediary *X* to control the species of interest *Y*. Variations in *X* due to extrinsic variability of the input are perfectly correlated to those in *Y*, and such knowledge can be leveraged to reduce these variations almost completely. However, this is not true for variations due to chemical reaction stochasticity. In the case of the decoupled production implementations, the variations in *X* due to this source of stochasticity are uncorrelated to those of *Y*. Any action by the circuits will result in noise amplification, and hence leads to an increase in the variance of *Y*. For the coupled production implementations, the variations in *X* are correlated to those in *Y*, but such correlation is not perfect due to the degradation of the species being decoupled. As such *X* acts as a “noisy sensor” of *Y*, and there is a limit to how much useful information content is in *X* and how aggressively it can be exploited. The best improvement comes from low expected abundance of *X* compared to *Y* (more “sensitive” sensor) and for small levels of effective gain.

Therefore, when trying to suppress both extrinsic and intrinsic sources of variation, there are tradeoffs to consider in both architectures since the best results occur at different gains (regulation strengths). In most applications, the more aggressive implementations would generally work best since the main source of variability tends to be extrinsic and that is where these circuits are most effective.

### A Circuit that Combines both FB and IFF Regulation Can Be More Effective in Dealing with Input Variability, but Is Just as Ineffective in Dealing with Chemical Reaction Stochasticity

Our work demonstrates that the combined circuit has an effective gain that is greater than the sum or product of the gains of the individual components. This allows for implementation of large effective gains even when high-gain implementation of separate IFF and FB components are infeasible, making higher adaptation to extrinsic variability possible. On the other hand the limitations in suppressing chemical reaction stochasticity do not rise from the inability to implement high gains circuits, but rather from the use of an intermediary in regulation. Indeed the optimal implementation for suppressing chemical reaction stochasticity has the same small effective gain as the optimal IFF and FB implementations.

Our analytical and computational analysis reveal that FB and IFF architectures, though at first glance can appear very different, can be considered as two sides of the same coin. They both have the capability to provide adaptation to changes in the input and some suppression of chemical reaction stochasticity through similar mechanism: exploiting the information contained in the proxy to control either the production or degradation of the species of interest. The main differences between the two lie in the ability to adapt to dynamic input fluctuations and the biological constrains in their implementations.

## Materials and Methods

Simulations were done using the SPSens software package [25] and the ode solver ode45 in MathWorks Matlab 8.1. The derivations of the transfer functions are shown in the Supporting Information.

## Supporting Information

S1 Text More details on derivation of results. (PDF)
