# Supplementary material for: Implementation Considerations, Not Topological Differences, Are the Main Determinants of Noise Suppression Properties in Feedback and Incoherent Feedforward Circuits
Source: PLoS Comput Biol. 2016 Jun 3;12(6):e1004958. doi: 10.1371/journal.pcbi.1004958 (PMC4892630; doi:10.1371/journal.pcbi.1004958)
Supplement: S1 Text — (PDF) [file pcbi.1004958.s001.pdf]

Implementation considerations, not topological differences, are the main determinants of noise suppression properties in feedback and incoherent feedforward circuits - Supporting Information

Gentian Buzi[1] and Mustafa Khammash[1*]

May 13, 2016

1. Control Theory and Systems Biology, Department of Biosystems Science and Engineering, ETH Zürich.

* Corresponding Author: Mustafa Khammash, mustafa.khammash@bsse.ethz.ch

Short Title: Noise suppression properties of feedback and incoherent feedforward circuits

1 Deterministic Models - Suppression of Input Fluctuations

For the FB models, both coupled and decoupled reactions schemes (Table 1) yield the same ODE model

$$\begin{aligned} \dot{x} &= f(u)g(x) - l_1 x \\ \dot{y} &= f(u)g(x) - l_2 y. \end{aligned} \tag{S1}$$

Similarly, both coupled and decoupled IFF schemes result in the following ODE model

$$\begin{aligned} \dot{x} &= f(u) - k_1 x \\ \dot{y} &= f(u) - k_{12} x y - k_2 y. \end{aligned} \tag{S2}$$

In order to compare the performance of the two models in a meaningful way, we normalize them using the following criteria:

For a given nominal value of the input $u = \bar{u}$,

1. the steady state values of Eq. (S2) and Eq. (S1) should be the same,
2. the production rates ("inflow") at steady state should be the same for both models.

Denote the steady state value of x and y at $u = \bar{u}$ by $\bar{x}$ and $\bar{y}$ respectively. The above normalization conditions imply

$$l_1 = k_1,\ l_2 = k_2 + k_{12}\bar{x},\ g(\bar{x}) = 1, \tag{S3}$$

$\bar{x} = \frac{f(\bar{u})}{k_1}$, and $\bar{y} = \frac{f(\bar{u})}{l_2} = \frac{k_1 \bar{x}}{k_2 + k_{12}\bar{x}}$. This normalizations will be applied to the rest of this section.

1.1 Sensitivity to small local perturbations in u

Let $y_{ss}(u)$ be the steady state concentration of y as a function of u. The sensitivity of y_{ss} with respect to changes in u near $\bar{u}$ for the FB is calculated to be

$$\begin{aligned} f(u)g(x_{ss}) - l_1 x_{ss} &= 0 \\ f(u)g(x_{ss}) - l_2 y_{ss} &= 0 \\ &\Downarrow \\ \frac{\partial x_{ss}}{\partial u}(u) &= -\frac{f'(u)g(x_{ss})}{f(u)g'(x_{ss}) - l_1} \\ &\Downarrow \\ \frac{\partial y_{ss}}{\partial u}(u) = \frac{l_1}{l_2}\frac{\partial x_{ss}}{\partial u}(u) &= \frac{g(x_{ss})}{g(x_{ss}) - x_{ss} g'(x_{ss})}\frac{f'(u)}{f(u)} y_{ss} \end{aligned}$$

and therefore at $u = \bar{u}$ ($x_{ss} = \bar{x}$, $y_{ss} = \bar{y}$, $g(x_{ss}) = g(\bar{x}) = 1$)

$$\frac{\partial y_{ss}}{\partial u}(\bar{u}) = \frac{1}{1 + \alpha_{fb}} \frac{f'(\bar{u})}{f(\bar{u})} \bar{y} \tag{S4}$$

where

$$\alpha_{fb} := -\bar{x} \frac{\partial g}{\partial x}(\bar{x}). \tag{S5}$$

In the case of the IFF, the sensitivity of y_{ss} with respect to changes in u near $\bar{u}$ is calculated to be

$$\frac{\partial y_{ss}}{\partial u}(\bar{u}) = \frac{1}{1 + \alpha_{ff}} \frac{f'(\bar{u})}{f(\bar{u})} \bar{y} \tag{S6}$$

where

$$\alpha_{ff} := \frac{\bar{x} k_{12}}{k_2} \tag{S7}$$

is the ratio of the mediated degradation of y by x ($k_{12}\bar{x}$) to the unmediated degradation of y (k_2). α_{ff} determines how much control x has over y, with larger values of α_{ff} (i.e., more control x has on y) improving

performance (less sensitive y_{ss} to fluctuations in u). Indeed, for $k_2 = 0$ ($\alpha_{ff} = \infty$) model (S2), studied in detail in Sontag [2010], displays perfect adaptation which is indicative of integral-like feedback (with integrator $\sigma = y - x$, the difference in concentrations between X and Y, and error $e = (k_1 - k_{12}y)\,x)$. This implies that for finite α_{ff} (i.e., $k_2 > 0$) the IFF can be viewed as leaky integral feedback.

We refer toα_{fb} as the effective feedback gain and α_{ff} as the effective feedforward gain. The motivation comes from the fact that α_{fb} and α_{ff} are closely related to the gains of the controller near the steady state. Indeed, if we let $\xi = [\xi_1, \xi_2]^T$ be small perturbations of $[x, y]^T$ near the steady state value $[\bar{x}, \bar{y}]$. The dynamics of (S1) near the steady state are given by

$$\dot{\xi} = A_{fb}\xi - \alpha_{fb}B_{fb}C_{fb}\xi \tag{S8}$$

where $A_{fb} = \begin{bmatrix} -l_1 & 0 \\ 0 & -l_2 \end{bmatrix}$, $B_{fb} = k_1\,[1,1]^T$, $C_{fb} = [1,0]$. This can be viewed as the closed loop dynamics of $\begin{pmatrix} A_{fb} & B_{fb} \\ C_{fb} & 0 \end{pmatrix}$ by a proportional negative feedback controler with gain α_{fb}. Similarly, the dynamics of (S2) near the steady state are given by

$$\dot{\xi} = A_{ff}\xi - \frac{\alpha_{ff}}{1+\alpha_{ff}}B_{ff}C_{ff}\xi \tag{S9}$$

where $A_{ff} = \begin{bmatrix} -k_1 & 0 \\ 0 & -\frac{\bar{x}}{\bar{y}}k_1 \end{bmatrix} = \begin{bmatrix} -l_1 & 0 \\ 0 & -l_2 \end{bmatrix}$, $B_{ff} = l_1\,[0,1]^T$, $C_{ff} = [1,0]$ (after the normalization S3 has been applied). This can be viewed as the closed loop dynamics of $\begin{pmatrix} A_{ff} & B_{ff} \\ C_{ff} & 0 \end{pmatrix}$ by a proportional negative feedback controller with gain $\frac{\alpha_{ff}}{1+\alpha_{ff}}$. Both α_{fb} and α_{ff} are a measure of the local strength of control of (S1) and(S2).

1.2 Steady-state dependency on u

$\frac{\partial y_{ss}}{\partial u}(\bar{u})$ is a local measure of how sensitive the steady state of the output of interest y is to samll changes in u near $\bar{u}$. This measure is a function of $\bar{u}$ and for each $\bar{u}$ we can get a picture of the overall sensitivity of the system to changes in u. Can we calculate directly the dependency of the steady state value of y to u? For the IFF, such calculation results in

$$y_{ss}(u) = \frac{f(u)}{k_2 + \frac{k_{12}}{k_1}f(u)} = \frac{f(u)}{k_2\left(1+\alpha_{ff}\frac{f(u)}{f(\bar{u})}\right)} = \frac{(1+\alpha_{ff})\,f(u)}{l_2\left(1+\alpha_{ff}\frac{f(u)}{f(\bar{u})}\right)}, \tag{S10}$$

where the normalization (S3) is applied. For the FB, in the general case, we can only get an implicit formula

$$\begin{aligned} ug(\frac{l_2}{l_1}y_{ss}) - l_2 y_{ss} &= 0 \\ x_{ss} &= \frac{l_2}{l_1}y_{ss} \end{aligned}$$

Let consider a special case when the inhibition function g is given by the ramp function g_R

$$g_R(x;\alpha_{fb}) := \max\left\{1+\alpha_{fb} - \alpha_{fb}\frac{l_1}{f(\bar{u})}x, 0\right\}. \tag{S11}$$

In this case

$$\begin{aligned} 1+\alpha_{fb} - \alpha_{fb}\frac{l_2}{f(\bar{u})}y_{ss} &= \frac{l_2}{f(u)}y_{ss} \\ &\Downarrow \\ y_{ss} &= \frac{(1+\alpha_{fb})\,f(u)}{l_2\left(1+\alpha_{fb}\frac{f(u)}{f(\bar{u})}\right)}. \end{aligned} \tag{S12}$$

This dependency of y_{ss} on u for the FB is is the same as that of the IFF for the same effective gain (eq. (S10)).

1.3 Attenuation of small time-varying disturbances in u - frequency domain analysis

So far, we have considered the dependence of the steady state value on constant perturbations of u. What happens when u is time varying? How does that affect y? For simplicity of notation, we take $f(u) = u$. Near the steady state, for $u(t) = \bar{u} + \delta(t)$ and small δ, the dynamics are given by

$$\begin{aligned} \dot{\xi} &= A_c \xi + B_\delta \delta \\ \zeta &= C_\delta \xi \end{aligned}$$

where$\xi = [\xi_1, \xi_2]^T$ are small perturbations of $[x, y]^T$ near $[\bar{x}, \bar{y}]$, $\zeta = \xi_2$, $B_\delta = [1, 1]^T$, $C_\delta = [0, 1]$, and

$$A_c = A_c^{fb} := A_{fb} - \alpha_{fb} B_{fb} C_{fb} = \begin{bmatrix} -l_1 - \alpha_{fb} l_1 & 0 \\ -\alpha_{fb} l_1 & -l_2 \end{bmatrix}$$

for FB and

$$A_c = A_c^{ff} := A_{ff} - \frac{\alpha_{ff}}{1 + \alpha_{ff}} B_{ff} C_{ff} = \begin{bmatrix} -l_1 & 0 \\ -\frac{\alpha_{ff}}{1+\alpha_{ff}} l_1 & -l_2 \end{bmatrix}$$

for FF. How the effect of $\delta(t)$ on the perturbations ζ of desired output y can more easily be expressed in the frequency domain

$$\zeta(s) = G(s)\delta(s)$$

where $\zeta(s)$, $\delta(s)$ are the Laplace transform of $\zeta(t), \delta(t)$ respectively, s is the frequency (Laplace) variable, and $G(s)$ is a trasfer function gine by

$$G(s) = G_{fb}(s) := C_\delta \left(sI - A_c^{fb}\right)^{-1} B_\delta = \frac{l_1 + s}{(l_2 + s)\left(l_1(1 + \alpha_{fb}) + s\right)} \tag{S13}$$

for FB, and

$$G(s) = G_{ff}(s) := C_\delta \left(sI - A_c^{ff}\right)^{-1} B_\delta = \frac{\frac{l_1}{1+\alpha_{ff}} + s}{(l_2 + s)(l_1 + s)} \tag{S14}$$

for IFF (with the normalizations (S3) applied).

Note that the sensitivity $\frac{\partial y_{ss}}{\partial u}(\bar{u})$ is given by $G(0)$ ($s = 0$ correspond to the response to constant δ). In general, higher gain pushes one of the poles of G (the value of s for which $G(s) \to \pm\infty$) toward $-\infty$ for the FB, but has no effect on the poles of G for the FF. On the other hand, higher gain pushes the zero of G (the value of s for which $G(s) = 0$) toward the origin for the FF, but it has no effect on the zero of G for the FB. The $\mathcal{H}_2$-norm of G defined as

$$\|G\|_{\mathcal{H}_2} := \left(\frac{1}{2\pi}\int_{-\infty}^{\infty} |G(i\omega)|\, d\omega\right)^{\frac{1}{2}},$$

is given by

$$\|G_{fb}\|_{\mathcal{H}_2}^2 = \frac{l_1 + l_2(1 + \alpha_{fb})}{2l_2(1 + \alpha_{fb})(l_2 + l_1(1 + \alpha_{fb}))}$$

and

$$\|G_{ff}\|_{\mathcal{H}_2}^2 = \frac{1 + \frac{l_1}{l_2(1+\alpha_{ff})^2}}{2(l_1 + l_2)}.$$

The$\mathcal{H}_2$-norm has a few interesting interpretations.

One interpretation is that its square is a measure of the "energy" of the transient to the impulse response, i.e.,

$$\|G_{fb}\|_{\mathcal{H}_2}^2 = \|\zeta\|_2^2 := \int_0^{\infty} |\zeta(t)|^2\, dt$$

when $\delta(t)$ is the Dirac delta function (whose effect is equivalent to perturbing the system away from the steady state). So the $\mathcal{H}_2$-norm is a measure of the transient deviations from the steady state.

Another interpretation is that if δ is given by white noise, then the square of the $\mathcal{H}_2$-norm is the variance of the output as $t \to \infty$ when $\delta(t)$ is white noise.

$$\|G_{fb}\|^2_{\mathcal{H}_2} = \lim_{t\to\infty} \text{var}\zeta(t).$$

In order for either system (FB or FF) to have good disturbance attenuation properties (i.e., minimize the effect of variations of $u(t)$ on the value of desired output $y(t)$), then $\mathcal{H}_2$-norm of G needs to be made small. In this respect, the FB architecture is better suited since this norm can be made arbitrarely small by choosing arbitrarely large gain, i.e.,

$$\lim_{\alpha_{fb}\to\infty} \|G_{fb}\|^2_{\mathcal{H}_2} = 0.$$

On the other hand, this is not true for the IFF architecture since

$$\lim_{\alpha_{ff}\to\infty} \|G_{ff}\|^2_{\mathcal{H}_2} = \frac{1}{2\,(l_1 + l_2)}.$$

This result is not surprising since the controller implemented by the IFF does not effect the poles of the system. Since the action of the controller has no effect on the behavior of x, and the "transient behavior" of uncontrolled x contributes to the transient behavior of y. Indeed $\|G_{ff}\,(\alpha_{ff} \to \infty)\|^2_{\mathcal{H}_2} = \frac{l_2}{l_1+l_2}\,\|G_{ff}\,(\alpha_{ff} = 0)\|^2_{\mathcal{H}_2}$, where $G_{ff}\,(\alpha_{ff} \to \infty)$ and $G_{ff}\,(\alpha_{ff} = 0)$ is the transfer function of a controller with infinite gain and zero gain respectively (i.e., maximum control and no control). For the FB, the controller controls both x and y, and is thus able to suppress the transient behavior of both species.

In conclusion, while the steady state behavior of both FB and IFF is the same for the same effectual gains, it is not true for the transient behavior. The FB architecture is better suited to deal with attenuating transient effects of disturbances.

1.4 IFFL circuit, non-catalytic effect of x

So far we have assumed that the role of x in the mediated degradation of y in the IFF is purely catalytic, i.e., x is not degraded as part of the reaction. What happens if the role of x is not always purely catalytic? Assume that the following reaction

$$X + Y \xrightarrow{k_{12}xy} \emptyset, \tag{S15}$$

takes place instead of reaction

$$X + Y \xrightarrow{k_{12}xy} X$$

with frequency a. Then the corresponding ODE is given by

$$\begin{aligned} \dot{x} &= f(u) - ak_{12}xy - k_1x \\ \dot{y} &= f(u) - k_{12}xy - k_2y. \end{aligned} \tag{S16}$$

If an isolated equilibrium point in the positive orthant exists, then we define the "load effect" of the non-catalytic degradation of y as $\rho := \frac{ak_{12}\bar{y}}{k_1+ak_{12}\bar{y}}$ (i.e., the ratio of the non-catalytic degradation of x, to the total degradation of x at steady state). The sensitivity of y_e with respect to changes in u near $\bar{u}$ is calculated to be

$$\frac{\partial y_e}{\partial u}\,(\bar{u}) = \frac{1}{1+\tilde{\alpha}_{ff}}\,\frac{f'(\bar{u})}{f(\bar{u})}\bar{y}, \tag{S17}$$

where $\tilde{\alpha}_{ff} = \alpha_{ff}(1-\rho)$. Notice that $\tilde{\alpha}_{ff} < \alpha_{ff}$, and larger the load ρ, the smaller the effective gain $\tilde{\alpha}_{ff}$ of the loaded circuit.

2 Stochastic Models - Suppression of Chemical Reaction Stochasticity

In this section we examine how the inherent stochasticity of the chemical reactions affects the ability of FB and IFF topologies to maintain a desired number of molecules of Y. In this section x and y are random variables that refer to the number of molecules of X and Y respectively. We study how different effective gain α_{fb} and α_{ff} change the noise properties of FBL and IFFL respectively, by looking at the steady state value of the variance of y.

2.1 Feedback Inhibition

Consider the case when for a given α_{fb}, the inhibition function g is parameterized by g_R

$$g_R(x;\alpha_{fb}) := \max\left\{g_L(x;\alpha_{fb}),0\right\}, \tag{S18}$$

where

$$g_L(x;\alpha_{fb}) := 1+\alpha_{fb}-\alpha_{fb}\frac{l_1}{f(\bar{u})}x.$$

I.e., g_R is a linearly decresing function of x for $x < x_0 := \frac{1+\alpha_{fb}}{\alpha_{fb}}\frac{f(\bar{u})}{l_1}$ and identically 0 for $x > x_0$ (Figure 3A, solid gray line). Notice that this choice of the inhibition function g is consistent with the definition of α_{fb} in Eq. S5. Let the set Γ be the set of all feedback gains for which x_0 is an integer, and consider only $\alpha_{fb} \in \Gamma$. Such set of α_{fb} is sufficient for our purpose of studying the noise properties of the FB architecture.

The stochastic models based on the coupled FB reaction scheme and the decoupled FB reaction scheme (Table 1), yield the following moment equation

$$\frac{d}{dt}\mathbf{m}_{XY} = L_F\mathbf{m}_{XY} + f(u)\mathbf{b}_F \tag{S19}$$

where $\mathbf{m}_{XY} = \left[\mathbb{E}\left[x\right],\mathbb{E}\left[y\right],\mathbb{E}\left[x^2\right],\mathbb{E}\left[xy\right],\mathbb{E}\left[y^2\right]\right]^T$,

$$L_F = \begin{bmatrix} -l_1 & 0 & 0 & 0 & 0 \\ 0 & -l_2 & 0 & 0 & 0 \\ l_1 & 0 & -2l_1 & 0 & 0 \\ 0 & 0 & 0 & -\left(l_1+l_2\right) & 0 \\ 0 & l_2 & 0 & 0 & -2l_2 \end{bmatrix},$$

$$\mathbf{b}_F = \begin{bmatrix} \mathbb{E}\left[g_R(x)\right] \\ \mathbb{E}\left[g_R(x)\right] \\ \mathbb{E}\left[\left(1+2x\right)g_R(x)\right] \\ b_{xy} \\ \mathbb{E}\left[\left(1+2y\right)g_R(x)\right] \end{bmatrix},$$

$b_{xy} = \mathbb{E}\left[\left(1+x+y\right)g_R(x)\right]$ for the coupled FB, and $b_{xy} = 0$ for the decoupled FB.

By Lemma 1 in the appendix, we have that for any $\alpha_{fb} \in \Gamma_{\bar{x}}$, $x \sim \mathcal{B}(x_0, \frac{\alpha_{fb}}{\alpha_{fb}+1})$ and therefore $\Pr\left(x > x_0\right) = 0$. This allows us to replace g_R in all the expectations in the moments equation (S19) by g_L (since $\Pr\left(x > x_0\right) = 0 \Rightarrow \mathbb{E}\left[x^i y^j g_R(x)\right] - \mathbb{E}\left[x^i y^j g_L(x)\right] = 0$). This allows us to solve

$$L_F\mathbf{m}_{XY} + f(u)\mathbf{b}_F = 0$$

exactly and get exact analytical solution for the steady state of the mean and variance of y. For the coupled FB we get

$$\mathbb{E}\left[y\right] = \frac{f(\bar{u})}{l_2} = \bar{y}$$

and

$$var(y) = \frac{1}{1+\alpha_{fb}}\bar{y} + P^{fb}_{proxy}$$

where

$$P_{proxy}^{fb} = \frac{\alpha_{fb}\left(\alpha_{fb} + \bar{x}/\bar{y}\right)}{\left(1+\alpha_{fb}\right)\left(\left(1+\alpha_{fb}\right) + \bar{x}/\bar{y}\right)}\bar{y} \tag{S20}$$

and $\bar{x} := \mathbb{E}[x]$. Notice that for each ratio $\frac{\bar{x}}{\bar{y}}$, the optimal gain is given by $\alpha_{fb}^{opt} = \sqrt{1+\bar{x}/\bar{y}}$, with corresponding variance

$$var^{opt}(y) = \frac{\left(2+\sqrt{1+\frac{\bar{x}}{\bar{y}}}\right)(x+y)y}{\left(1+\sqrt{1+\frac{\bar{x}}{\bar{y}}}\right)\left(\bar{x}+\bar{y}+\sqrt{1+\frac{\bar{x}}{\bar{y}}}\bar{y}\right)}$$

. The reduction of the variance by the best feedback strategy is greater for smaller the ratios $\bar{x}/\bar{y}$, with the best case scenario ($\bar{x}/\bar{y} \to 0$) reducing the variance by 25% ($var(y, \alpha_{fb} \to 1, \bar{x} \to 0) = \frac{3}{4}\bar{y}$).

For the decoupled FB, the mean remains the same and the steady state variance is evaluated as

$$var(y) = \tfrac{1}{1+\alpha_{fb}}\bar{y} + P_{proxy}^{fb} + P_{decouple}^{fb}$$

where

$$P_{decouple}^{fb} = \frac{\alpha_{fb}}{1+\alpha_{fb}+\bar{x}/\bar{y}}\bar{y}. \tag{S21}$$

2.2 Incoherent Feedforward

The moment equations of the stochastic model based on the coupled and decoupled IFF reaction scheme (Table 1) for $u = \bar{u}$ yield $\mathbb{E}_{ss}[x] = \bar{x}$, $var_{ss}(x) = \bar{x}$, $\bar{x} = \frac{f(\bar{u})}{k_1}$.

$$\frac{d}{dt}\mathbf{m}_{XY} = L_I\mathbf{m}_{XY} + \mathbf{b}_I \tag{S22}$$

$$L_I = \begin{bmatrix} -k_1 & 0 & 0 & 0 & 0 \\ 0 & -k_2 & 0 & -k_{12} & 0 \\ 2f(u)+k_1 & 0 & -2k_1 & 0 & 0 \\ f(u) & f(u) & 0 & -k_1-k_2 & 0 \\ 0 & 2f(u)+k_2 & 0 & k_{12} & -2k_2 \end{bmatrix}$$

$$B_I = f(u)\begin{bmatrix} 1 \\ 1 \\ 1 \\ b_4 \\ 1 \end{bmatrix} + \begin{bmatrix} 0 \\ 0 \\ 0 \\ -k_{12}\mathbb{E}\left[x^2y\right] \\ -2k_{12}\mathbb{E}\left[xy^2\right] \end{bmatrix}$$

where $b_4 = 1$ for the coupled case and $b_4 = 0$ for the decoupled case. Note that x is simply a birth death process, and therefore has a Poisson stationary distribution with mean and variance given by $\bar{x} = \frac{f(\bar{u})}{k_1}$.

Special case - no regulation In the case $k_{12} = 0$, $k_2 > 0$ (no regulation, $\alpha_{ff} = 0$), the mean of the stationary distribution of y is given by $< y >= \frac{f(\bar{u})}{k_2} = \bar{y}$ and the variance $var(y) = \bar{y}$ (both coupled and decoupled implementations give the same values, y is simply a birth death process).

General case For $k_2 > 0$, $k_{12} > 0$, using derivative matching Singh and Hespanha [2011] we approximate $\mathbb{E}\left[x^2y\right]$ and $\mathbb{E}\left[xy^2\right]$ by

$$\mathbb{E}\left[x^2y\right] = \frac{\mathbb{E}\left[x^2\right]}{\mathbb{E}[y]}\left(\frac{\mathbb{E}[xy]}{\mathbb{E}[x]}\right)^2$$

$$\mathbb{E}\left[xy^2\right] = \frac{\mathbb{E}\left[y^2\right]}{\mathbb{E}[x]}\left(\frac{\mathbb{E}[xy]}{\mathbb{E}[y]}\right)^2$$

and solve $L_I\mathbf{m}_{XY} + \mathbf{b}_I = 0$ for this approximations to get expressions for $< y^2 >$, the second moment of the stationary distibution of y,

$$< y^2 > \quad = \quad \frac{1+\bar{z}\alpha_{ff}}{1+\bar{z}^2\alpha_{ff}}\left(\bar{y}+1\right)\bar{y}$$

where $\alpha_{ff} = \frac{k_{12}\bar{x}}{k_2}$ and $\bar{z} := \frac{<xy>}{\bar{x}\bar{y}}$ is the positive solution of

$$m(z) := \alpha_{ff}\left(1+\bar{x}+\bar{y}\right)z^2 + \left(\bar{x}+\bar{y}-\alpha_{ff}\left(b_4+\bar{x}+\bar{y}\right)\right)z - \left(b_4+\bar{x}+\bar{y}\right) = 0$$

For $b_4 = 1$, $\frac{<xy>}{\bar{x}\bar{y}} > 1, \forall \alpha_{ff} > 0$ and therefore $var(y) < \bar{y}$. So for the coupled production, regulation reduces the variance. $\frac{<xy>}{\bar{x}\bar{y}} > 1$ implies that there is a positive correlation between x and y, i.e. fluctuations in x carry information about the fluctuations in y and regulation can use this information to suppress such fluctuations. Aggressive action (regulation) on such imperfect information can have adverse effect and eventually loose the benefit of regulation ($\frac{<xy>}{\bar{x}\bar{y}} \to 1$ and $var(y) \to \bar{y}$ as $\alpha_{ff} \to \infty$).

Indeed, algebraic manipulations show that the gain that reduces the variance the most is given by

$$\alpha_{ff} = \frac{\bar{x}+\bar{y}}{1+\bar{x}+\bar{y}} < 1$$

with corresponding second moment

$$< y^2 >= \frac{1}{2}\frac{1+\bar{x}+\bar{y}+\sqrt{\left(\bar{x}+\bar{y}\right)^2+\bar{x}+\bar{y}}}{1+\bar{x}+\bar{y}}\left(\bar{y}+1\right)\bar{y}$$

which is monotone decreasing in $\bar{x}$. For $\bar{x}$ very small

$$var(y) \quad \underset{\bar{x}\to 0}{\longrightarrow} \quad \frac{1}{2}\left(1-\bar{y}+\sqrt{\bar{y}^2+\bar{y}}\right)\bar{y}$$

which means a reduction of variance by about 25% for $\bar{y}$ (varying from $var(y) = \frac{\sqrt{2}}{2}\bar{y}$ for $\bar{y} = 1$ to $var(y) = 0.75\bar{y}$ as $\bar{y} \to \infty$).

In the case of the decoupled IFF ($b_4 = 0$), $\frac{<xy>}{\bar{x}\bar{y}} < 1, \forall \alpha_{ff} > 0$ and therefore $var(y) > \bar{y}$. Therefore for the decoupled IFF realizations, any regulation increases the variance (amplifies the stochastic noise). In fact, the stronger the gain, the larger the amplification. $\frac{<xy>}{\bar{x}\bar{y}} < 1$ implies that there is negative correlation between x and y, i.e., any regulation pushes x and y in opposite direction and therefore increases the variance (regulation is acting on stochastic noise, fluctuations in x do not carry any useful information about fluctuations in y).

Special case - infinite gain In the case $k_2 = 0$ (infinite gain α_{ff}) the explicit expressions for the variance and the penalty terms are calculated. The mean of the stationary distribution of y for the coupled implementation is given by $< y >= \frac{k_1}{k_{12}} = \bar{y}$ and the variance $var(y) = \bar{y}$. There is no improvement of the variance of y compared to the no regulation ($k_{12} = 0$) instance. For the decoupled IFF, we get

$$\begin{aligned} \bar{y} \quad &= \quad \frac{k_1 - k_{12}\bar{x}}{2k_{12}} + \frac{1}{2}\sqrt{\left(\bar{x}+\frac{k_1}{k_{12}}\right)^2 + 4\frac{k_1}{k_{12}}} \\ var(y) \quad &= \quad \left(\frac{k_{12}}{k_1}\bar{y} - 1 + \frac{k_{12}}{k_1}\right)\bar{y}^2 \end{aligned}$$

($\bar{x} = f(\bar{u})/k_1$). The same parameter values used in the coupled case would result in a higher mean for the decoupled case. In order for any comparison to be meaningful, we set both coupled and decoupled models to have the same $\bar{y}$. To this end, the mediated consumption rate constant for the decoupled model $\tilde{k}_{12}$ should be

$$\tilde{k}_{12} = k_{12}\left(1+\frac{k_{12}}{k_1+\bar{x}k_{12}}\right)$$

The rate constant $\tilde{k}_{12}$ is larger than k_{12}, implying that faster mediated degradation of y is required for the decoupled system to maintain the same expected value of y. Using this rate constant, the adjusted variance is

$$var(y) \quad = \quad \bar{y} + \frac{\bar{y}+1}{1+\bar{x}/\bar{y}}$$

So there is a decoupled production penalty of $P^{ff}_{decouple} = \frac{\bar{y}+1}{1+\bar{x}/\bar{y}}$. So the most aggressive regulation can more than double the variance in the worst case scenario (very small average population of X) and increase the variance only by a little in the best case scenario (very large average population of X).

2.3 Comparing the Different Architectures

Both FB and IFF circuits respond in a similar manner to chemical reaction stochasticity and achieve comparable reductions in steady state variance (no more than 25% in all but a few extreme scenarios). Direct comparison of coupled FB and IFF reductions for optimal values of gains, shows that for average molecule counts of X and Y that are no smaller than 2, the FB realizations achieve larger reductions than the IFF counterparts. The larger the average population of X, the more FB outperforms the IFF. Notice also that the optimal gain for the FB is larger than the optimal gain for the IFF.

3 Concurrent Suppression of Both Sources of Variability

In this section we consider both sources of noise simultaneously. We rely on the analytical results of the previous sections and numerical simulations to derive general principles on how both architectures suppress both sources of noise and what are the best strategies for doing so.

So far we have showed that if the stochasticity of the chemical reactions can be ignored (i.e., the dynamics of the circuit can be faithfully represented by their macroscopic representation), regulation always reduces fluctuations due to input noise and for strong enough levels of regulation completely suppress such fluctuations. On the other hand, if the input noise can be ignored and the primary source of noise is the randomness of the chemical reactions, regulation (especially strong regulation) is not always beneficial with the best case scenario only improving the variance by 25%. However such reductions of the extrinsic and intrinsic noise cannot be not achieved simultaneously by the same circuit (i.e., such improvements require different effective gains which for each circuit realization are hard encoded by the reaction propensities). There seems to be a tradeoff on how aggressively the circuit should regulate the production (for the FB) or the degradation (for the IFF) of Y .

We illustrate this idea with an example. Consider three different FB circuit realizations:

1. no feedback regulation ($\alpha_{fb} = 0$, $g(x) \equiv 1$)
2. maximum level of regulation (very large effective gain, $\alpha_{fb} \to \infty$), coupled production
3. maximum level of regulation ($\alpha_{fb} \to \infty$), decoupled production.

For the last two realizations, the feedback inhibition function g given by (S18). Let the input u be a random variable distributed according to a Poisson distribution (such as the stationary distribution of a birth death process) with mean $\bar{u}$, and let $f(u) = au + b$. For the first realization (no feedback)

$$var(y) \quad = \quad \frac{C+b}{l_2} + \frac{C^2}{l_2^2}\frac{1}{\bar{u}},$$

where $C := a\bar{u}$. For constant C and b (which guarantees constant average inflow $f(u)$), smaller $\bar{u}$ implies larger total variance as the result of a larger input noise component (the second term). This is to be expected since smaller $\bar{u}$ means that Poisson variable u becomes more "noisy". Note that for this realization, both coupled and decoupled implementations yield the same $var(y)$.

The second FB realization (coupled, very strong feedback) results in a smaller variance, $var(y) = \frac{C+b}{l_2}$, for any finite $\bar{u}$. This is a result of strong feedback completely suppressing the variance term due to randomness

in u but not changing the variance term due to randomness of the chemical reactions. So for the coupled production, strong feedback is prefered to no feedback. We will later show that, depending on the noise characteristics of u, more moderate feedback yields a bigger reduction in the overall variance.

For the third FB realization, (coupled, very strong feedback), strong feedback again completely suppresses the variance term due to randomness in u but it also doubles the variance term due to randomness of the chemical reactions resulting in $var(y) = 2\frac{C+b}{l_2}$. So this realization is preferable to no feedback only if $\bar{u} < \frac{C^2}{l(b+C)}$, i.e. the input noise level is above a specific threshhold.

In general, for both FB and IFF circuits the best regulation strategy is dependent on the relative dominance of the terms in the variance decomposition. Figure 4 shows numerical simulation results for different circuit realizations and different input noise scenarios. As the input noise level is high, the extrinsic noise term is dominant and stronger regulation is preferred. For low levels of input noise, the intrisic noise term becomes dominant and small levels of regulations are preferred especially for the decoupled realizations.

4 Combining FB and IFF into a Single Circuit

The reaction scheme is the same as the IFF scheme of Table 1 with that change that the rate of production reactions is given by $f(u)g(x)$ and we follow the same normalization rules for g. This results in the following ODE model

$$\begin{aligned} \dot{x} &= f(u)g(x) - k_1 x \\ \dot{y} &= f(u)g(x) - k_{12}xy - k_2 y. \end{aligned} \tag{S23}$$

We define the "effective gain" $\alpha_{comb} := \alpha_{fb} + \alpha_{ff} + \alpha_{fb}\alpha_{ff}$, and note that both equations 1 and 3 hold.

Near the steady state, for $u(t) = \bar{u} + \delta(t)$ and small δ, the dynamics are given by

$$\begin{aligned} \dot{\xi} &= A_c \xi + B_\delta \delta \\ \zeta &= C_\delta \xi \end{aligned}$$

where$\xi = [\xi_1, \xi_2]^T$ are small perturbations of $[x, y]^T$ near $[\bar{x}, \bar{y}]$, $\zeta = \xi_2$, $B_\delta = [1, 1]^T$, $C_\delta = [0, 1]$, and

$$A_c = \begin{bmatrix} -l_1 - \alpha_{fb} l_1 & 0 \\ -\frac{\alpha_{comb}}{1+\alpha_{ff}} l_1 & -l_2 \end{bmatrix}.$$

Using Linear noise approximation, the contribution of each chemical reaction i to the overall variace of y can be caluculated using the $\mathcal{H}_2$-norm of the transfer function G_i

$$G_i(s) := [0, 1] (sI - A_c)^{-1} B_i \sqrt{\omega_i} \tag{S24}$$

where I is the identity matrix, B_i is the stoichiometry of reaction i, and ω_i is the propensity of reaction i.

Appendix

Given the reaction scheme,

$$R_1 : \emptyset \xrightarrow{fg(x)} x, \qquad R_2 : x \xrightarrow{k_1 x} \emptyset,$$

where $g(x) = g_R(x)$

$$g_R(x; \alpha) := \max\left\{1 + \alpha - \frac{\alpha}{\bar{x}} x, 0\right\},$$

and $\bar{x} = \frac{f}{k_1}$. Define

$$x_0 := \left\lceil \frac{1+\alpha}{\alpha} \bar{x} \right\rceil = n.$$

Notice that $\Pr\{x > x_0\} = 0$, since the propensity function of reaction R_1 is zero for $x > x_0$. The matrix form of the chemical master equation (CME) for the first $x_0 + 1$ states is given by,

$$\dot{\mathbf{p}} = A\mathbf{p},$$

where $\mathbf{p} = [p_0, \ldots, p_n]^T$ and $A = -diag\{[s_0, \cdots, s_n], 0\} + diag\{[r_1, \cdots, r_n], 1\} + diag\{[m_0, \cdots, m_{n-1}], -1\}$ where $\forall i$, $s_i = m_i + r_i$, $m_i = fg(i)$, $r_i = k_1 i$, and $n = x_0$. $diag\{\mathbf{v}, k\}$ is the matrix with the elements of $\mathbf{v}$ in the kth diagonal (the rest of the entries are zero); $k = 0$ is the main diagonal. The steady state value of $\mathbf{p}$ satisfies $A\mathbf{p} = 0$, and the entries satisfy

$$\frac{p_i}{p_{i-1}} = \frac{m_{i-1}}{r_i}, \tag{S25}$$

and are given by

$$p_k = \frac{\prod_{i=1}^{n-k} r_{n-i+1}}{\prod_{i=1}^{n-k} m_{n-i}} p_n, \tag{S26}$$

subject to

$$1 = \sum_{k=0}^{n} p_{n-k} = \left(\sum_{k=0}^{n} \frac{\prod_{i=1}^{k} r_{n-i+1}}{\prod_{i=1}^{k} m_{n-i}} \right) p_n. \tag{S27}$$

Lemma 1. If $\frac{1+\alpha}{\alpha}\bar{x}$ is an integer then at steady state $x \sim \mathcal{B}\left(x_0, \frac{\bar{x}}{x_0}\right)$ (binomial distribution).

Proof. $x_0 = n = \frac{1+\alpha}{\alpha}\bar{x} \Rightarrow \bar{x} = \frac{\alpha}{1+\alpha}n$, and from Eq. (S26) we get

$$p_k = \frac{\prod_{i=1}^{n-k} (n-i+1)}{\prod_{i=1}^{n-k} i\alpha} p_n = \left(\begin{array}{c} n \\ n-k \end{array} \right) \left(\frac{1}{\alpha} \right)^{n-k} p_n \tag{S28}$$

and

$$\begin{aligned} 1 = \sum_{k=0}^{n} p_k &= p_n \sum_{k=0}^{n} \left(\begin{array}{c} n \\ n-k \end{array} \right) \left(\frac{1}{\alpha} \right)^{n-k} = \left(\frac{1}{\alpha} + 1 \right)^n p_n \\ &\Downarrow \\ p_n &= \left(\frac{\alpha}{1+\alpha} \right)^n = \left(\frac{\bar{x}}{x_0} \right)^{x_0}. \end{aligned}$$

Substituting for p_n in Eq. (S28), we get for any $1 \leq k \leq n$

$$\begin{aligned} p_k &= \left(\begin{array}{c} n \\ n-k \end{array} \right) \left(\frac{1}{\alpha} \right)^{n-k} \left(\frac{\alpha}{1+\alpha} \right)^n \\ &= \left(\begin{array}{c} n \\ n-k \end{array} \right) \left(\frac{\alpha}{1+\alpha} \right)^k \left(\frac{1}{1+\alpha} \right)^{n-k} \end{aligned}$$

which is exact the k-th coefficient $q_{k,n}$ of $\mathcal{B}\left(n, \frac{\alpha}{1+\alpha}\right) = \mathcal{B}\left(x_0, \frac{\bar{x}}{x_0}\right)$ and so

$$x \sim \mathcal{B}\left(n, \frac{\alpha}{1+\alpha}\right) = \mathcal{B}\left(x_0, \frac{\bar{x}}{x_0}\right). \tag{S29}$$

□

Lemma 2. *If* $\frac{1+\alpha}{\alpha}\bar{x}$ *is not an integer, then at steady state* $\Pr\{x = x_0\} \leq \left(\frac{\alpha}{\alpha+1}\right)^{x_0}$.

Proof. Define $\tilde{x} := \frac{\alpha}{1+\alpha}x_0$, and define

$$\tilde{g}_R(x; \alpha) := \max\left\{1 + \alpha - \frac{\alpha}{\tilde{x}}x, 0\right\}.$$

It is clear that $\tilde{x} > \bar{x}$ and $\tilde{m}_x = f\tilde{g}_R(x) \geq fg_R(x)m_x$, $\forall x$. From Eq. (S27),

$$\begin{aligned} \left(\sum_{k=0}^{n} \frac{\prod_{i=1}^{k} r_{n-i+1}}{\prod_{i=1}^{k} m_{n-i}}\right) p_n &= \left(\sum_{k=0}^{n} \frac{\prod_{i=1}^{k} r_{n-i+1}}{\prod_{i=1}^{k} \tilde{m}_{n-i}}\right) \tilde{p}_n \\ &\Downarrow \\ p_n &\leq \tilde{p}_n \end{aligned}$$

where $\tilde{p}_n$ is probability $x = n$ for the model where R_1 has propensity $f\tilde{g}_R(x)$. From Eq. (S29) we have $\tilde{p}_n = \left(\frac{\alpha}{1+\alpha}\right)^{x_0}$, and therefore $p_n = \Pr\{x = x_0\} \leq \left(\frac{\alpha}{1+\alpha}\right)^{x_0}$. □

References

A. Singh and J. P. Hespanha. Approximate moment dynamics for chemically reacting systems. *IEEE Trans. on Automat. Contr.*, 56(2):414–418, Feb. 2011.

E.D. Sontag. Remarks on feedforward circuits, adaptation, and pulse memory. *Systems Biology, IET*, 4(1): 39–51, 2010. ISSN 1751-8849. doi: 10.1049/iet-syb.2008.0171.

	k_2	0.09	0.1	0.2	0.3	0.4	0.5	0.6	0.7	0.8	0.9	1
Fig. 3	Coupled k_{12}	0.0182	0.0180	0.0160	0.0140	0.0120	0.0100	0.0080	0.0060	0.0040	0.0020	0
	Decoupled k_{12}	0.0184	0.0182	0.0161	0.0141	0.0121	0.0101	0.0080	0.0060	0.0040	0.0020	0
Fig. 4	k_{12}	0.0909	0.0899	0.0797	0.0697	0.0596	0.0496	0.0396	0.0297	0.0197	0.0099	0

Table A: k_2 and k_{12} numerical values used in numerical simulations in Fig. 3 and 4 . k_2 and k_{12} were chosen so that they result in the corresponding α_{ff} and $\bar{y}$ using the formulas for α_{ff} and $\bar{y}$ in the main text.

	feedback		incoherent feedforward	
	coupled	decoupled	coupled	decoupled
degradation reactions	$X \xrightarrow{5x} \emptyset,\ Y \xrightarrow{y} \emptyset$		$X \xrightarrow{5x} \emptyset,\ Y \xrightarrow{k_2 y} \emptyset$<br>$X+Y \xrightarrow{k_{12}xy} X$	
production reactions	$U \xrightarrow{f(u)g(x)} X+Y$	$U \xrightarrow{f(u)g(x)} X,$<br>$U \xrightarrow{f(u)g(x)} Y$	$U \xrightarrow{f(u)} X+Y$	$U \xrightarrow{f(u)} X,$<br>$U \xrightarrow{f(u)} Y$
U dynamics	$\emptyset \xrightarrow{1}, U\ U \xrightarrow{u/\bar{u}} \emptyset$		$\emptyset \xrightarrow{1}, U\ U \xrightarrow{u/\bar{u}} \emptyset$	

Table B: Feedback and incoherent feedforward model parameters for simulations in Fig 4. The propensities of the reactions are shown above the reaction arrows, and $g(x) = \frac{11}{1+10(x/50)^h}$, $f(u) = 50u/\bar{u}$. For IFF, k_2 and k_{12} values used in simulation are shown in Table S1.
